# Exposure to Early Life Adversity and Interpersonal Functioning in Attempted Suicide

**DOI:** 10.3389/fpsyt.2020.552514

**Published:** 2020-09-17

**Authors:** Mia Rajalin, Tatja Hirvikoski, Ellinor Salander Renberg, Marie Åsberg, Jussi Jokinen

**Affiliations:** ^1^ Department of Clinical Sciences, Umeå University, Umeå, Sweden; ^2^ Centre for Psychiatry Research, Stockholm, Stockholm County Council, Stockholm, Sweden; ^3^ Paediatric Neuropsychiatry Unit, Center of Neurodevelopmental Disorders at Karolinska Institutet (KIND), Department of Women’s and Children’s Health, Karolinska Institutet, Stockholm, Sweden; ^4^ Habilitation & Health, Stockholm County Council, Stockholm, Sweden; ^5^ Department of Clinical Sciences, Danderyd Hospital (KI DS), Stockholm, Sweden; ^6^ Department of Clinical Neuroscience, Karolinska Institutet, Stockholm, Sweden

**Keywords:** interpersonal problems, suicide attempt, early life adversity, adverse childhood experiences, Inventory of Interpersonal problems, Karolinska Interpersonal Violence Scale, suicide – attempted, mood disorder

## Abstract

**Background:**

Early life adversity (ELA) may lead to an increased risk for mental health problems including suicidal behavior. ELA alters biological stress systems that affect behavior and control within the individual that in turn will affect interpersonal behavior. Strained relations and interpersonal conflicts leading to rejection and isolation have been shown to be factors for suicidal behavior. Difficulties in interpersonal relationships are a common reason for seeking help in psychiatric care. In the present study, we examined relationship between different types of interpersonal problems and adverse childhood experiences in patients with a recent suicide attempt.

**Method:**

The study included 181 recent suicide attempters. We assessed early life adversity and specific interpersonal problems by using the Karolinska Interpersonal Violence Scale and the Inventory of Interpersonal problems respectively.

**Results:**

Suicide attempters with high levels of early life adversity expressed a more socially avoidant, non-assertive, and exploitable personal style even after adjustment for comorbidities with personality disorder and substance use disorder.

**Conclusions:**

Patients with a recent suicide attempt with high levels of early life adversity tend to isolate themselves, of being introvert, and having difficulties to open up and confide in others. They report low self-confidence and self-esteem and problems with feeling and expressing anger. These behaviors complicate interaction with others and make establishment of solid relationships more difficult. In regards to detection of suicidal communication and treatment of suicidal patients, this may lead to misinterpretations and difficulties to fully benefit from treatment given or for professionals to provide the appropriate treatment. Clinicians should closely investigate the presence of early life adversity in suicidal patients and pay attention to their personal style and their difficulties in interpersonal exchange.

## Introduction

According to data from the World Health Organization (1), suicide accounts for approximately 800,000 deaths each year. Moreover, the impact of suicide does not limit itself to the closest family and friends, in fact, many others in the society are affected, like health care providers, workplace colleagues, and neighbors (2). Suicide remains a true challenge for the world public health systems and should be a priority in every nation. Many mechanisms are involved in the development of suicidal behavior and a better understanding of these factors is crucial for successful suicide prevention.

Extensive research on risk factors both at the population and individual levels has been conducted and psychiatric disorders particularly have a strong impact on suicide rates as well as previous suicide attempt, a family history of suicidal behavior, and childhood sexual abuse. Precipitating factors in vulnerable individuals are considered to result in psychological changes, including feeling hopeless and burdensome, which may lead in turn to social isolation. One of the more widely studied risk factors for suicide is exposure to violence. Early experiences of both emotional and physical abuse are recognized as both biological and psychological risk factors leading to different negative outcomes in adulthood, including a higher suicide risk (3–6). In our previous study (5) focusing on the family history of suicide (FHS) and early life adversity (ELA) on suicide risk, we found that ELA was a predictor for suicide in women without regard to FHS. In men with both FHS and ELA, the risk for suicide was clearly heightened compared to those with only FHS. ELA was consequently a determinant for suicide in both men and women.

For individuals with fulfilled criteria for Posttraumatic Stress Disorder (PTSD), there is a strong association with suicidal behavior (7, 8). However, even in the subclinical population with PTSD symptoms, the association with suicidal behavior is strong (9). Furthermore, ELA is a known factor in interpersonal dysfunctional patterns expressed in personality and behavior (10–12) leading to a research focus on the individual predisposition for suicidal behavior (13). Several personality and psychological traits have been associated with suicidal behavior. For example, impulsivity and instability in interpersonal relationships are often found in the patients with borderline personality disorder (BPD) (14–16). Other interpersonal aspects, like lack of social connectedness, and the feeling of being a burden to others are known risk factors for suicide as they will increase the tendency of isolation (17, 18).

Problematic interpersonal behaviors are common among suicidal patients, (19) with shown deficits in active interpersonal problem solving (20). Interpersonal problems like avoidance, social isolation and submissiveness are common in patients with affective disorders, especially major depressive disorder (21). In fact, rejection and isolation related to interpersonal conflicts appeared as the main triggers of suicidal behavior in several studies on life events preceding a completed suicide (22–24).

To date, only few studies have investigated the relationship between early life adversity and interpersonal functioning, and to our knowledge very little research has been done so far with focus on attempted suicide (25). This study aims to investigate associations between early life adversity and interpersonal problems both assessed with structured instruments in patients with a recent suicide attempt.

## Materials and Methods

### Study Setting

Patients having clinical follow-up after a suicide attempt at the Suicide Prevention Clinic at the Karolinska University Hospital, Stockholm, Sweden were invited to participate in two clinical studies which took place between the years of 1993–2005.

### Participants

One hundred eighty-one suicide attempters (113 women and 68 men) were recruited according to following inclusion criteria: a recent suicide attempt, an age of 18 years or older and a command of Swedish language. Suicide attempt was defined as any non-fatal, self-injurious behavior with some intent to die (1). Patients with following diagnoses- schizophrenia spectrum psychosis, intravenous drug abuse, intellectual disability, and dementia were excluded. Trained psychiatrists diagnosed participants with the research version of SCID-I according to DSM III and DSM IV. To establish any occurrence of Axis-II diagnosis, the SCID-II interviews were performed by trained clinical psychologists. Almost all (91%) of the patients had at least one current Axis-I diagnosis. The most prevalent primary diagnosis was mood disorder (75%) while anxiety disorder and adjustment disorder each were present in 5% of patients. Substance use disorder as a primary Axis I diagnosis was present in 3%, one individual had anorexia nervosa and another had an unspecified psychiatric disorder. One third of the patients met criteria for a personality disorder. The most prevalent were a personality disorder not otherwise specified (39%) and borderline personality disorder (35%) followed by dependent personality disorder (11%), and avoidant personality disorder (9%). Six percent of the patients fulfilled criteria for antisocial personality disorder.

### Assessments

The Karolinska Interpersonal Violence Scale (KIVS) subscale B “Victim of violence” in childhood 6–14 years was used to assess adverse childhood experience (26). Statements about being a victim of violence is rated between (0), no exposure to interpersonal violence, (1), occasional slaps, occasional fights of no importance, (2), bullied for short period of time, occasionally exposed to physical punishment, (3), repeatedly bullied and/or beaten in school and in home environment, (4), bullied throughout childhood, regularly beaten by parent or other adult, sexual abuse, and (5), repeatedly exposed to violent behavior that results in severe bodily harm, repeated severe sexual abuse. Trained professionals performed assessments using a semi-structured interview. A high inter-rater reliability of the scale has been reported (26) and KIVS has been used in suicide research (27, 28). The KIVS subscale B “Victim of violence” in childhood has been validated using the Childhood trauma Questionnaire in a study of patients with hypersexual disorder (29). The KIVS exposure to interpersonal violence as a child showed a significant correlation with the CTQ-SF total score indicating that KIVS subscale measuring exposure to violence in childhood has validity as a clinical tool assessing early life adversity (29).

Inventory of Interpersonal Problems (IIP), a 64-item self-report inventory with a well-established validity and reliability (0.78), (30) was used to assess the most evident interpersonal problems (31). The IIP has been validated in Sweden (32). Eight subscales describing each different interpersonal problems (new denomination in parenthesis) are included in the IIP: Domineering (Domineering/Controlling); Vindictive (Vindictive/Self-centered); Cold (Cold/Distant); Social avoidant (Social inhibited); Non-assertive (Non-assertive); Exploitable (Overly Accommodating); Overly nurturant (Self-sacrificing), and Intrusive (Intrusive/Needy). The subjects respond to two forms of statements “it is hard for me to…” and “these are things I do too much or too often” of how they usually cope with distressing interpersonal behaviours. The answers are rated on a Likert scale ranging from 0 (not at all) to 4 (extremely).

The scale Domineering captures lack of control and aggressing others. “I try to change people too much” is an example of a statement in this scale. Scoring high in the scale Vindictive implies problems with vindictive thoughts, frustration and anger: “it’s hard for me to put someone else’s needs before my own”. A high value in the scale Cold implies difficulties to connect to others with examples of items like “I keep other people at a distance too much” and “it’s hard for me to get along with other people”. The Social avoidant scale describes a rejective and introvert personal style with statements like “it’s hard for me to show my feelings” and “it’s hard for me to socialize with other people”. High scores in the scale Social avoidant indicates feelings of anxiety, shame or shyness when interacting with other people. The Non-assertive scale reflects low self-confidence and low self-esteem. “It’s hard for me to be firm when I need to be” is an example of a statement in the Non-assertive scale: Individuals with high scores in this scale doubt themselves and avoid expressing their needs for fear of criticism. High scorers in the Exploitable scale struggle to please others and report problems with anger expression. Examples of statements are “it is hard for me to let other people know when I’m angry” and “I let other people take advantage of me too much”. In the scale Overly nurturant a characteristic is to have difficulties with boundaries. “I put other people’s needs before my own too much” is one example of the inclination to try too hard. Finally, the scale Intrusive with items like “I tell personal things to other people too much” and “it’s hard for me to stay out of other people’s business” captures the individual’s problems with boundaries.

The total score, translated to a normative T-score, reflects the individual’s general level of interpersonal problems in relation to the normal population. The scores on eight scales describe the specific problematic domains and type of interpersonal problems. An ipsatized T-score that relates to the individuals’ own problem level can also be used. The scores of IIP scale may thus be used to assess a patient’s level of interpersonal problems, to compare different groups, or as a measurement of interpersonal problems before and after clinical treatment.

### Statistical Methods

We used Shapiro Wilks test to evaluate skewness and kurtosis of the distributions. Correlational analyses were used to determine associations between the exposure to interpersonal violence as a child and the eight subscales of IIP. Pearson’s r or Spearman rho were applied for parametric or non-parametric correlation analyses. Student’s t-test and the Kruskal-Wallis’ test were used to assess group differences (suicide attempters with and without comorbid personality disorder or substance abuse, respectively) in continuous variables.

Based on the results of the bivariate analyses, standard multiple regression analyses were conducted to determine whether early life adversity was associated with specific type of interpersonal problems adjusted for comorbidities with personality disorder and substance use disorder. All statistical tests were two-tailed.

The p value was set at <0.05. We used the Statistical Package JMP 9.0.3 software, SAS Institute Inc., Cary, NC, USA.

## Results

### Level of Interpersonal Problems

The mean T-scores are shown in **Table 1** (n = 162; range for subscales 49–61). Patients with a comorbid personality disorder had significantly higher scores in all eight subscales (all p values < 0.01) compared to patients without a comorbid personality disorder. Comorbidity of substance use disorder was associated with significantly higher scores in domains Non-assertive (p = 0.042) and Social avoidant (p = 0.043).

**Table 1:** IIP-ratings in suicide attempters (n = 162), T-score mean, standard deviation (SD), range.

Rating	IIP total	
	T score Mean (SD)	Range
Domineering	49 (12)	38–96
Vindictive	56 (16)	38–114
Cold	55 (13)	39–96
Social avoidant	56 (15)	35–104
Non-assertive	55 (16)	31–98
Exploitable	59 (15)	30–100
Overly nurturant	61 (15)	32–103
Intrusive	52 (12)	34–85

IIP, Inventory of Interpersonal Problems.

### Early Life Adversity and Interpersonal Problems in Adulthood

Patients with a comorbid personality disorder or a comorbid substance abuse disorder reported significantly higher exposure to interpersonal violence as a child compared to patients without comorbidity (Z = 4.12, p < 0.0001; Z = 3.99, p < 0.0001).

**Table 2** shows correlations between exposure to interpersonal violence in childhood and interpersonal problems in adulthood in patients with a recent suicide attempt. The domains Social avoidant, Non-assertive, Exploitable, Cold, and Overly nurturant were significantly, positively correlated with exposure to interpersonal violence during childhood. Multiple regressions of the significant scales of IIP (Social avoidant, Non-assertive, Exploitable, Cold, and Overly nurturant) as a dependent variable, exposure to interpersonal violence as a child as well as comorbid personality disorder and substance use diagnosis as independent variables, were conducted.

**Table 2:** Correlations (Spearman’s ρ) between the KIVS exposure to interpersonal violence as a child and IIP in suicide attempters.

	Overly nurturant	Intrusive	Domineering	Vindictive	Cold	Socially avoidant	Non-assertive	Exploitable
KIVS	0.16*	0.13	0.03	0.13	0.18*	0.31***	0.25**	0.22**

p, p-value; IIP, Inventory of Interpersonal Problems; KIVS, Karolinska Interpersonal Violence Scale.

*p < 0.05, **p < 0.01, ***p < 0.001.

The overall models were significant for three of IIP domains- Social avoidant, Non-assertive and Exploitable with adjusted R^2^ = 0.05–0.09, meaning that the models explained 5–9% of the variance of these IIP subscales, **Table 3**.

**Table 3:** Exposure to interpersonal violence, comorbid personality disorder, and substance use disorder as predictors of IIP Social avoidance, Non-assertive, and Exploitable in suicide attempters.

Social avoidance	t ratio	*p* value
KIVS exposure as a child	3.10	0.0022
Comorbid Personality disorder	2.27	0.024
Substance use disorder	0.27	0.78
Non-assertive	t ratio	*p* value
KIVS exposure as a child	2.61	0.0097
Comorbid Personality disorder	1.47	0.14
Substance use disorder	0.81	0.42
Exploitable	t ratio	*p* value
KIVS exposure as a child	2.67	0.0081
Comorbid Personality disorder	1.23	0.22
Substance use disorder	0.02	0.98

**Figure 1:**
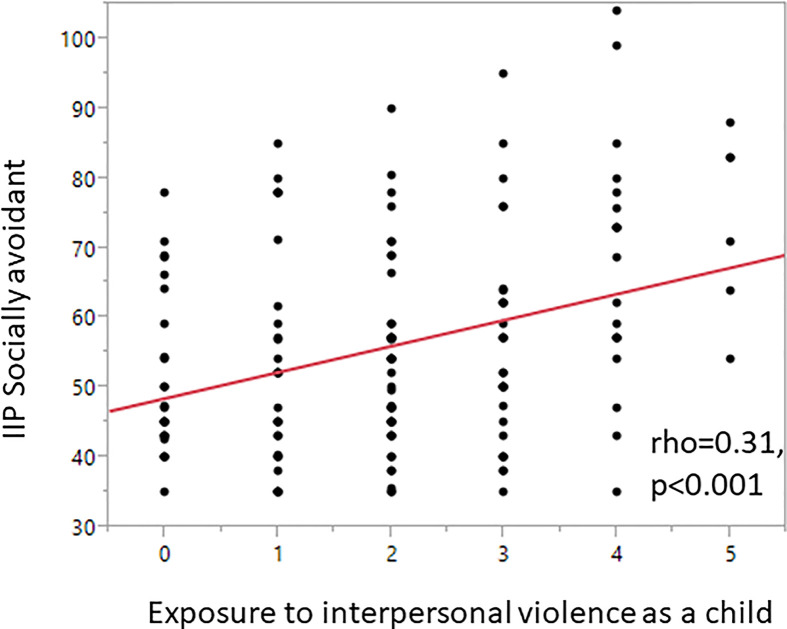
Higher IIP ratings in the subscale Social avoidant were associated with higher scores of KIVS exposure to interpersonal violence as a child (rho = 0.31, p < 0.001).

The standardized values of t-ratio for exposure to interpersonal violence in childhood (between 2.61–3.10), indicated that higher scores in childhood interpersonal violence exposure were associated with higher scores of three IIP scales Social avoidant, Non-assertive and Exploitable. **Figure 1** shows correlation between exposure to interpersonal violence as a child and Social avoidance.

## Discussion

In this study of patients in treatment after a suicide attempt, we wanted to investigate the relationship between early life adversity and interpersonal problems. The main finding was that suicide attempters with high levels of early life adversity expressed a more socially avoidant, non-assertive, and exploitable personal style even after adjustment for comorbidities with personality disorder and substance use disorder. Compared to the normative sample, the T-scores were higher for suicide attempters in all IIP-scales. However, none of the eight scales reached the level of severe interpersonal difficulties in IIP which is defined in the literature scoring two standard deviations above the mean score. Generally, the suicide attempters scored one standard deviation higher, and the scores varied more compared to the normative sample. In line with the hypothesis, patients with a comorbid personality disorder had higher scores in every domain of interpersonal problems, while a comorbid substance use diagnosis was associated to significantly higher scores in the domains Social avoidant and Non-assertive in group comparisons.

Patients with high levels of ELA had significantly higher scores on the scales Social avoidant, Non-assertive, and Exploitable when taking into account comorbidity with personality disorders and substance use disorder. These findings suggest an impaired ability in social interaction, and in how to express feelings and thoughts when with others. The difficulties may manifest as a tendency to keep to oneself. Further, patients who reported high levels of ELA scored lower self-confidence and self-esteem and reported problems with feeling and expressing anger in efforts to try hard to please other people. Behaviors like these may jeopardize establishment of strong relationships since they are often regarded as aversive by the environment. Furthermore, in regards to detection of suicidal communication and treatment of suicidal patients, this may lead to misinterpretations and difficulties to fully benefit from treatment given or for professionals to provide the appropriate treatment. In clinical practice the patient may seem difficult to cooperate with, which leads to risk for disrupted contact. Stable and strong personal relationships may protect for suicidal crisis, during which the ability to seek and accept help from others is of importance (33). In long term, the patient may lose important relationships due to these interpersonal problems accentuating social isolation and feelings of hopelessness.

IIP has a negative correlation with The Hogan Personality Inventory (HPI) that is a measurement of normal personality qualities that describe how one relates to others when at one´s best (34). The total score on IIP has the highest correlation with the HPI scale Adjustment—with measures to which extent a person appears self-content and at ease as well as open to feedback from others. The IIP scale that has the highest negative correlation to Adjustment is Social Avoidant which then would suggest the opposite, a tense and self-critical person that resists to feedback.

When it comes to ELA, KIVS captures not only many types of maltreatment but also duration or repetitive exposures. However, due to the construct of the scale, it is not possible to investigate a specific type of victimization like sexual abuse which prior studies often have focused on. The KIVS subscale exposure to interpersonal violence in childhood has been validated using the Childhood trauma questionnaire, a gold standard instrument to measure early life adversity (29). CTQ total score as well as subscales measuring physical and emotional abuse showed significant correlations with KIVS exposure to interpersonal violence in childhood (29). However, many research studies have reported that multi-victimization is frequent among children and adolescents (35, 36). Bullying has been associated with suicidal behavior (37) and with depression and isolation (38). In connection to this, the subjective perception of social support has been shown to be a key element in suicidal behavior (10, 39, 40). Early risk factors play a role in attempted and completed suicide in epidemiological studies (41), as well as personality traits (42).

Isolation, either real or just the subjective experience of desolation, is an important factor in suicidal behavior, and a focus on social skills training in the suicidal patient is important. The specific interpersonal patterns related to ELA that appeared in suicide attempters in this study may have effect on their ability to have long-lasting, stable relationships.

Strengths of this study include the large sample of suicide attempters and the careful clinical characterization. A limitation is that we did not unfortunately have information concerning how many patients were eligible to participate during the whole study period.

Our results indicate that suicide preventive efforts should include programs at population level toward the detection and elimination of childhood abuse and broad support related to the issue, effective and thorough assessment of risk individuals in health care and their ability to connect to others and seek social support. Therapeutic interventions should consider the different aspects of patients’ interpersonal functioning and aim to increase the individual´s ability to maintain positive relationships.

## Data Availability Statement

The raw data supporting the conclusions of this article will be made available by the authors, without undue reservation.

## Ethics Statement

The studies involving human participants were reviewed and approved by: The Regional Ethical Review Board in Stockholm approved of the study protocols (Dnr 93-211 & Dnr 00-194). The patients/participants provided their written informed consent to participate in this study.

## Author Contributions

Recruiting participants: JJ, MR. Performed the psychiatric interviews: JJ, MR. Formulating the problem and hypothesis: MR, JJ, TH, ER, and MÅ. Analyzed data: MR, JJ, TH. Wrote the paper: MR, JJ. Approved the final version of paper: MR, JJ, TH, ER, and MÅ.

## Funding

Funding for this study was provided by the Swedish Research Council (Project numbers: 5454; K2009-61P-21304-04-4; K2009-61X-21305-01-1) and through a regional agreement between Umeå University and Västerbotten County Council (ALF) and by grants provided by the Stockholm County Council (ALF).

## Conflict of Interest

The authors declare that the research was conducted in the absence of any commercial or financial relationships that could be construed as a potential conflict of interest. 
